# Supplementary material for: From sequence to enzyme mechanism using multi-label machine learning
Source: BMC Bioinformatics. 2014 May 19;15:150. doi: 10.1186/1471-2105-15-150 (PMC4229970; doi:10.1186/1471-2105-15-150)
Supplement: Additional file 2 — Java code of ml2db. Additional file ml2db_code.tar.gz contains the Java source code to run the multi-label machine learning experiments and save the results to database. The code’s Javadoc is included. [file 1471-2105-15-150-S2.zip › additional file 2/ml2db/ml2db/doc/main/ml2db/mulan/learn/traintest/TrainTestExperimenter.html]

TrainTestExperimenter


JavaScript is disabled on your browser.


- Overview
- Package
- Class
- Use
- Tree
- Deprecated
- Index
- Help

- Prev Class
- Next Class

- Frames
- No Frames

- All Classes

- Summary:
- Nested |
- Field |
- Constr |
- Method

- Detail:
- Field |
- Constr |
- Method


main.ml2db.mulan.learn.traintest

## Class TrainTestExperimenter

- java.lang.Object
- - main.ml2db.mulan.learn.crosseval.CrossExperimenter
  - - main.ml2db.mulan.learn.traintest.TrainTestExperimenter

- Direct Known Subclasses:
  :   AlExperimenter, TrainTestExperimenterSerialized

  ---

    

  ```
  public class TrainTestExperimenter
  extends CrossExperimenter
  ```

  Using Mulan (multi-label machine learning), this class initialises and
  executes Train-Test evaluation (two arff records are needed, for the training
  and testing files) (calling `TrainTestEvaluator`). Initialisation
  includes verifying input (train arff, test arff and train xml files) and
  checking output locations (for saving evaluation statistics and predictions
  to file and database).

  Version:
  :   29 Sep 2010

  Author:
  :   Luna De Ferrari ldf(at)st-andrews.ac.uk

- - ### Constructor Summary

    Constructors

    Constructor and Description

    `TrainTestExperimenter(DbManagers dbMan,
    int trainArffRecordId,
    int testArffRecordId,
    MulanLearner learner,
    java.lang.String resultsDirectory)`

    `TrainTestExperimenter(IdMultiLabelInstances trainSet,
    IdMultiLabelInstances testSet,
    DbManagers dbManagers,
    MulanLearner learner,
    java.lang.String resultsDirectory)`
  - ### Method Summary

    Methods

    | Modifier and Type | Method and Description |
    | --- | --- |
    | `void` | `evaluateAndSave(boolean predict, boolean saveTrueNegatives)` |
    | `TrainTestEvaluator` | `getEvaluator()` |
    | `TrainTestInputManager` | `getInputManager()` |
    | `TrainTestEvaluator` | `getNewEvaluator()` |
    | `TrainTestOutputManager` | `getOutputManager()` |
    | `static void` | `main(java.lang.String[] args)` |
    | `static void` | `run()` |
    | `void` | `setInputManager(IdMultiLabelInstances trainSet)` |
    | `void` | `setInputManager(int trainArffRecordId, DbManagers dbMan)` |
    | `void` | `setInputManager(int trainArffRecordId, DbManagers dbMan, TrainTestExperimenter trainTestExperimenter)` |
    | `void` | `setOutputManager(uk.ac.standrews.utils.main.database.DbManager resultsDbManager, java.lang.String resultsFolder, uk.ac.standrews.utils.main.database.Table table)` |

    - ### Methods inherited from class main.ml2db.mulan.learn.crosseval.CrossExperimenter

      `evaluateAndSave, getLearner, getTestArffId, getTrainArffId, run, setTestArffId`
    - ### Methods inherited from class java.lang.Object

      `equals, getClass, hashCode, notify, notifyAll, toString, wait, wait, wait`

- - ### Constructor Detail


    - #### TrainTestExperimenter

      ```
      public TrainTestExperimenter(DbManagers dbMan,
                           int trainArffRecordId,
                           int testArffRecordId,
                           MulanLearner learner,
                           java.lang.String resultsDirectory)
      ```


    - #### TrainTestExperimenter

      ```
      public TrainTestExperimenter(IdMultiLabelInstances trainSet,
                           IdMultiLabelInstances testSet,
                           DbManagers dbManagers,
                           MulanLearner learner,
                           java.lang.String resultsDirectory)
      ```
  - ### Method Detail


    - #### evaluateAndSave

      ```
      public void evaluateAndSave(boolean predict,
                         boolean saveTrueNegatives)
      ```


    - #### getEvaluator

      ```
      public TrainTestEvaluator getEvaluator()
      ```


    - #### getInputManager

      ```
      public TrainTestInputManager getInputManager()
      ```

      **Overrides:**
      :   `getInputManager` in class `CrossExperimenter`


    - #### getNewEvaluator

      ```
      public TrainTestEvaluator getNewEvaluator()
      ```


    - #### getOutputManager

      ```
      public TrainTestOutputManager getOutputManager()
      ```

      **Overrides:**
      :   `getOutputManager` in class `CrossExperimenter`


    - #### setInputManager

      ```
      public void setInputManager(IdMultiLabelInstances trainSet)
      ```

      **Overrides:**
      :   `setInputManager` in class `CrossExperimenter`


    - #### setInputManager

      ```
      public void setInputManager(int trainArffRecordId,
                         DbManagers dbMan)
      ```

      **Overrides:**
      :   `setInputManager` in class `CrossExperimenter`


    - #### setInputManager

      ```
      public void setInputManager(int trainArffRecordId,
                         DbManagers dbMan,
                         TrainTestExperimenter trainTestExperimenter)
      ```


    - #### setOutputManager

      ```
      public void setOutputManager(uk.ac.standrews.utils.main.database.DbManager resultsDbManager,
                          java.lang.String resultsFolder,
                          uk.ac.standrews.utils.main.database.Table table)
      ```

      **Overrides:**
      :   `setOutputManager` in class `CrossExperimenter`


    - #### main

      ```
      public static void main(java.lang.String[] args)
      ```


    - #### run

      ```
      public static void run()
      ```


- Overview
- Package
- Class
- Use
- Tree
- Deprecated
- Index
- Help

- Prev Class
- Next Class

- Frames
- No Frames

- All Classes

- Summary:
- Nested |
- Field |
- Constr |
- Method

- Detail:
- Field |
- Constr |
- Method
